# EasyLCMS: an asynchronous web application for the automated quantification of LC-MS data

**DOI:** 10.1186/1756-0500-5-428

**Published:** 2012-08-11

**Authors:** Sergio Fructuoso, Ángel Sevilla, Cristina Bernal, Ana Belén Lozano, José Luis Iborra, Manuel Cánovas

**Affiliations:** 1 Department of Biochemistry and Molecular Biology B and Immunology, Faculty of Chemistry, University of Murcia, Apdo. Correos 4021, 30100, Murcia, Spain; 2 Inbionova Biotech S.L., Edif. CEEIM, Universidad de Murcia, Campus de Espinardo, 30100, Murcia, Spain

**Keywords:** Metabolomics, LC-MS, Data handling, Asynchronous, Quantification, Quantitation, Automated calibration, Web application, LC distortion

## Abstract

**Background:**

Downstream applications in metabolomics, as well as mathematical modelling, require data in a quantitative format, which may also necessitate the automated and simultaneous quantification of numerous metabolites. Although numerous applications have been previously developed for metabolomics data handling, automated calibration and calculation of the concentrations in terms of μmol have not been carried out. Moreover, most of the metabolomics applications are designed for GC-MS, and would not be suitable for LC-MS, since in LC, the deviation in the retention time is not linear, which is not taken into account in these applications. Moreover, only a few are web-based applications, which could improve stand-alone software in terms of compatibility, sharing capabilities and hardware requirements, even though a strong bandwidth is required. Furthermore, none of these incorporate asynchronous communication to allow real-time interaction with pre-processed results.

**Findings:**

Here, we present EasyLCMS (http://www.easylcms.es/), a new application for automated quantification which was validated using more than 1000 concentration comparisons in real samples with manual operation. The results showed that only 1% of the quantifications presented a relative error higher than 15%. Using clustering analysis, the metabolites with the highest relative error distributions were identified and studied to solve recurrent mistakes.

**Conclusions:**

EasyLCMS is a new web application designed to quantify numerous metabolites, simultaneously integrating LC distortions and asynchronous web technology to present a visual interface with dynamic interaction which allows checking and correction of LC-MS raw data pre-processing results. Moreover, quantified data obtained with EasyLCMS are fully compatible with numerous downstream applications, as well as for mathematical modelling in the systems biology field.

## Findings

### Background

**Figure 1:**
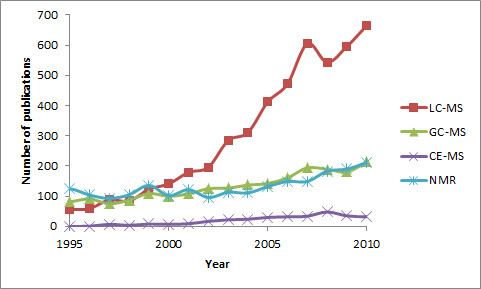
**Number of publications related to analytical platforms used in targeted metabolomics.** Number of publications related with quantification in the metabolomics area. Search criteria in ISI were ‘mass spectrometry’ AND quant* AND metab* AND ‘liquid chromatography’ for LC-MS, ‘gas chromatography’ for GC-MS or ‘capillary electrophoresis’ for CE-MS. For nuclear magnetic resonance, the term ‘mass spectrometry’ was substituted for ‘NMR’.

Metabolomics is an -omic science dedicated to studying low molecular mass organic compounds present in biological organisms. Although high molecular weight polymers such as DNA or proteins are discarded, small polymers such as oligopeptides can be included in the term [1]. Traditionally, two different approaches have been followed for metabolite analysis—targeted (or quantitative) and non-targeted, which is dedicated to identifying more than quantification of metabolites. For targeted metabolomics, several analytical platforms have been applied, including NMR, GC-MS, CE-MS, LC-MS and LC-UV. In contrast to what is generally accepted [2], GC-MS is not the most commonly used technology for quantitative analysis in the metabolomics field (Figure 1); rather, the most widespread technology is LC-MS, which started to be extensively used several years ago. The main reason for this is that most biological compounds are charged rather than volatile, and so must be derivatised for GC-MS.

In terms of raw data analysis, several platforms have been developed for the quantitative approach, although most of them were designed for GC-MS or focus on proteomics data [3]. Moreover, the GC-MS platform peak alignment among samples is based on retention indices to minimise shifts and to increase reproducibility. On the other hand, LC-MS peak alignment is more problematic, since it is based on retention time, which usually presents non-linear deviation during analysis (an example is shown in the Supplementary Material) [4-6]. Yet, this effect is not taken into account in the majority of applications designed for GC-MS [7]. Additionally, libraries for specific quantifier ions, including retention times or indexes, are needed when GC-MS applications are employed. These lists are usually generated manually (without any visual confirmation) or using the software AMDIS [8]. However, AMDIS does not provide metabolite IDs based on popular data bases such as KEGG or PubChem.

In addition to the above, two approaches have been adopted: stand-alone and web-based. In general, stand-alone are usually software applications which are executed in the user’s computer. A particular example of stand-alone applications is vendor software; these have several limitations [9], and do not clearly establish which methods are used for data handling, contrary to what should be the case [10].

Web-based applications perform computation in central servers using a web browser as interface, meaning that (i) they are easily accessible on different platforms, (ii) no or minimum installation is needed, (iii) fixes or updates are automatically incorporated, (iv) results can be shared among scientists in different locations and (v) more powerful servers can be used for hosting, thus increasing calculation capabilities [11]. On the other hand, web applications can be slower due to multiple and simultaneous access to the same resources, and because file uploading depends on the internet connection bandwidth. Moreover, analysts should be able to interact with the processed results, for example, to check correct peak assignment. However, classical web applications have limitations related to user interaction, since once information or action is provided, the web page needs to be reloaded due to the use of synchronous communication with the sever. In this web application model, user interaction is based on sending information to the server and then waiting until the server has processed the information and returns a new web page. In consequence, very few web applications are available for targeted metabolomics, and even fewer can interact directly with raw MS data, since classical web applications could not handle MS data interaction in real time. On the other hand, web applications based on asynchronous communication with the server could allow a single-page interface which neither needs to wait for the server nor to reload the complete web page; therefore, these would behave similarly to stand-alone applications.

This is possible because user interaction is performed through a software engine which can be independent of the communication with the server (detailed information can be found in [12]). Furthermore, user interaction can be enriched with such web applications, allowing real-time interaction, and consequently improving MS data visua-lisation. Among the different possibilities for enriched visualisation, Silverlight [13] has been selected, since it allows the querying and visualisation of large datasets (for a recent example see the update of the Frequency of INherited Disorders database (FINDbase) [14]).

Recently, downstream web applications able to analyse metabolomics data, including MetaboAnalyst [15], MSEA [16], ProMetra [17] or metaP-server [18], have appeared. These can use quantified data in terms of μmol, but metabolites need to be identified according to a database (Pubchem, KEGG, HMDB, etc.). Moreover, mathematical modelling requires quantified data for validation and parameter calculation [19]. To obtain data in this format from LC-MS experiments, several software/applications should be integrated into a pipeline, requiring strong knowledge in data handling [20]. Moreover, pre-processing of LC-MS data is nowadays in the development process, since several steps in the data processing need to be improved, such as peak detection or alignment algorithms; therefore, misalignment, as well as errors in the peak detection, have been found in all the software recently analysed [6,20].

EasyLCMS is a web application designed to fulfil the connection between raw LC-MS data and targeted metabolomics quantification, automatically retrieving metabolite IDs from popular databases and recent non-linear algorithms [4] to carry out peak alignment. Furthermore, the quantification process is completely integrated, requiring minimal knowledge of data handling or programming. Moreover, EasyLCMS implements an easy web-based interface with asynchronous communication to allow chromatogram visualisation and analyst interaction in real time.

## Implementation

**Figure 2:**
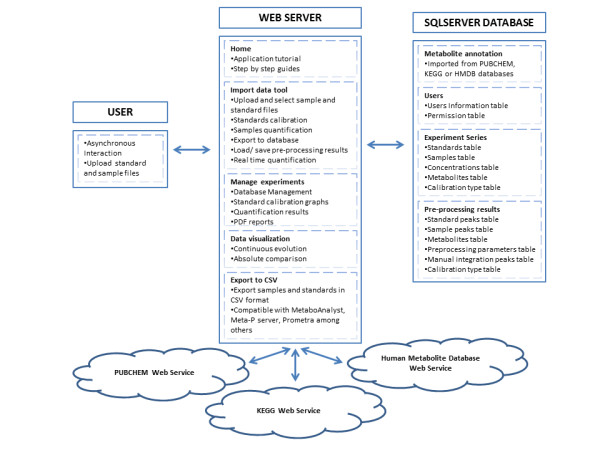
**Structural overview of the EasyLCMS web application.** EasyLCMS consists of three interacting layers: (**i**) web-interface, (**ii**) web-server processing modules and (**iii**) SQL-database. Details can be found in the EasyLCMS application tutorial.

**Figure 3:**
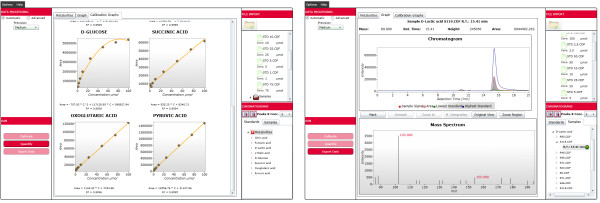
**Screenshots of the EasyLCMS web application.** The quantification process is completely integrated and automated in the EasyLCMS platform. However, manual intervention is allowed at all steps to check and correct automatic results when needed.

**Figure 4:**
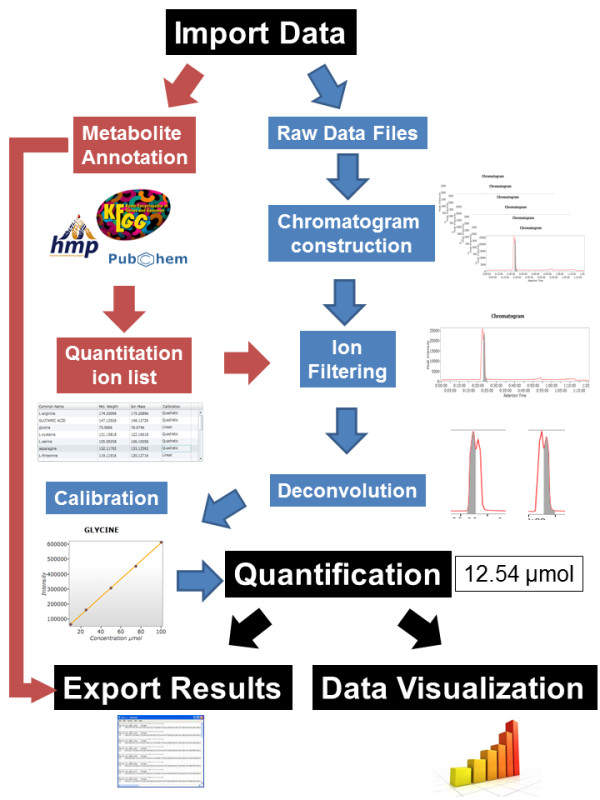
**Overview of the EasyLCMS data workflow.** The quantification workflow schema is represented. Detailed instructions are provided in the main text and EasyLCMS application tutorial.

EasyLCMS is a web application with three interacting layers, as represented in Figure 2 and detailed in the application tutorial. Screenshots of this application are shown in Figure 3. The LC-MS data workflow comprises the steps described in the following sections and represented in Figure 4.

### Metabolite annotation and data import

EasyLCMS is able to search for metabolite annotation in three different databases (Pubchem, HMDB and KEGG) using a compound name. The molecular weight of the compound is automatically obtained and converted into a quantification ion, taking into account the ionisation method, although this can also be manually established. This step cannot be carried out so easily in GC-MS, since derivatisation is usually required, or MS^n^ since m/z depends on the fragmentation pattern. Moreover, EasyLCMS checks for interconnectivity among the databases and is able to import additional IDs from different databases to avoid multiple searches. For example, if a metabolite is found in HMDB, KEGG and/or Pubchem, IDs are automatically downloaded. If an internal standard is used, it can be easily and directly defined in the table, in which case, standard and sample areas are normalised as previously established [9] (see Supplementary Material). The quantification ion list can be saved for future experiments. Raw data files can also be uploaded to the server using the following currently supported formats: mzML (1.0 and 1.1), mzXML (2.0, 2.1 and 3.0) and NetCDF. Zip files are also supported. For other formats, several converters are available [21].

### Data processing

Data processing (Figure 4) is carried out in three steps: (i) raw data filtering, (ii) chromatogram construction and (iii) deconvolution. For raw data filtering, the Savitzky–Golay smoothing filter can be employed. Afterwards, the MS spectrum is converted into m/z and intensity lists using a centroid algorithm which detects all data points above the specified noise level. Then, consecutive m/z values are connected to form continuous chromatograms (chromatogram construction). At this point, previously established quantification ions are filtered to increase the speed of processing by avoiding the subsequent steps in every single chromatogram. Finally, the remaining chromatograms are divided into individual peaks (deconvolution) by an algorithm which identifies local minima in the chromatogram as border points between peaks. All the data processing algorithms have been developed by the MZmine 2 application crew [4]. To simplify data processing, three configurations using pre-established parameters have been chosen based on the optimisation of peak detection and minimisation of the time needed. Each step of data processing can be defined for advanced users.

### Calibration

The peak area is the parameter usually taken in LC to establish a mathematical relationship between the metabolite concentration and the signal of the instrument. The mathematical relationship between the peak area (A) and the concentration (C) or calibration curve can be linear or non-linear. EasyLCMS allows several types of calibration curves, namely (i) linear (A = a_0_ + a_1_·C), (ii) logarithmic (A = a_0_ + a_1_·Ln(C)), (iii) power (A = a_0_ C^a1^), (iv) exponential (A = a_0_·a_1_^C^), (v) quadratic polynomial (A = a_0_ + a_1_·C + a_2_·C^2^) and (vi) cubic polynomial (A = a_0_ + a_1_·C + a_2_·C^2^+ a_3_·C^3^), where a_0_, a_1_, a_2_ and a_3_ are the regression parameters. Standards samples of known concentrations are required to calculate the regression parameters and to construct the calibration curve, which will be used to determine the unknown concentration of the metabolites in samples. Although the theoretical minimum number of standard samples is generally two, with exception of quadratic polynomial and cubic polynomial which are three and four, respectively, six non-zero standard samples covering the expected range of concentration are highly recommended [22]. Replicates of standard samples are not required.

To start the calibration process, standard concentrations and raw data files should be added. The first time that a calibration process is carried out, the retention time of every metabolite must be provided by selecting a peak on the interactive chromatogram visualisation, which can be done in any standard sample. For the remaining standards and samples, the application suggests peaks candidates based on recent alignment algorithms [4], although the automatic selection can be manually changed afterwards. This step needs only be carried out once for the same analytical method; after this, the platform can be left unattended. With this procedure, writing tables or files with m/z and retention times is avoided and peaks are selected by visual confirmation. To the best of our knowledge, featuring online chromatogram visualisation in web applications has been performed previously [2,23], but none of these application are able to represent the peak area used for quantification or allow manual integration. The calibration results can be pre-visualised at this point.

### Quantification

As mentioned above, EasyLCMS suggests peak candidates for samples using the RANSAC alignment algorithm [4], which performs well in cases of non-linear deviation in retention times, as in LC-MS [4,6]. However, candidates can be also manually selected using visualisation of the chromatogram; moreover, quantification is performed in real time, and thus it is performed simultaneously as a peak is selected. In this or previous steps, peak selections can be saved for subsequent modification. Once the peaks are selected, the area under the peak are utilised to calculate sample concentrations using the linear or non-lineal regressions of the standard curves generated in the previous step with standard samples of known concentrations. Prior to the quantification step, EasyLCMS automatically suggests which regression has the best fit based on regression coefficients.

### Data visualisation

EasyLCMS allows visualisation of the results by bar diagrams (discrete data) or representation versus a given condition (continuous data).

### Export results

The quantitative metabolomics export format is usually in the form of comma-separated values (CSV) files with several columns, where the first column is used to provide the compound IDs. EasyLCMS can export quantitative results in this format using IDs from the major databases (Pubchem, KEGG and HMDB), since they are compatible with numerous web applications or software tools for subsequent analysis. For example, MSEA [16] can use almost every ID format, while ProMetra [17] and MetaP-Server [18] use KEGG IDs. The quantitative results generated with EasyLCMS are totally compatible with these applications.

## Results

### Applications and comparison with other applications

To quantify metabolites, the first option is normally the manufacturers’ software (Chemstation, Xcalibur, etc.). However, these applications have strong deficiencies [9]; for example, total ion chromatogram (TIC) is generally used for automated purposes, while using specific mass ions requires manual intervention. Therefore, they are time-consuming and clearly not suitable for automated simultaneous quantification. Although several software tools and web applications have been developed recently for targeted metabolomics data management, only a few are sufficiently specialised for HPLC-MS, for example, stand-alone applications such as MAVEN [24], MZmine 2 [4], XCMS [25] (with a recent online version) or the web service metaP-Server [18], among others. Some stand-alone applications designed for GC-MS can also be used for LC-MS raw data, including ADAP [26], MET-IDEA [27] and MetaQuant [9], or the web application MetabolomeExpressProject [2]. Surprisingly, almost all of these finish the quantification procedure at the point at which relative areas or heights from the compound peaks are provided (MetaQuant is the only exception). Therefore, manual intervention will be necessary to complete regression analysis for calibration using statistics software such as MS-Excel or SigmaPlot [7], especially if nonlinear regression is needed. To our knowledge, the only free application able to carry out unattended automated quantification in terms of μmol is MetaQuant, which is able to perform quantitative analysis including nonlinear regressions. However in MetaQuant, (i) metabolic IDs from common databases cannot be automatically retrieved, (ii) only netCDF and CSV formats are allowed, (iii) quantifier ions are manually added and (iv) no adjustment of retention time is performed for alignment, although the precision of peak alignment is reduced for HPLC-MS, as previously reported [4-6]. EasyLCMS improves the described applications since it is able to (i) automatically quantify metabolic samples without requiring additional statistics software for calibration, (ii) import several MS file formats (NetCDF, mzXML and mzML), (iii) search for metabolite names in the principal databases (HMBD, KEGG and Pubchem) to obtain all the IDs from these databases simultaneously, (iv) take into account LC-MS retention time drift and (v) allow analysts to interact in every step of the quantification process.

### Validation of the EasyLCMS platform

Two experiments were carried out in order to perform the validation of EasyLCMS. Firstly, the intracellular amino acid content of the human cell line CCL-159 in a batch reactor was measured using an internal standard. Secondly, the time course evolution of two different human cell lines—CCL-159 (S) and CCL-159R (R)—cultivated in batch reactors were analysed to quantify 29 metabolites (intracellular as well as extracellular). In this case, no internal standard was used to check the reliability of EasyLCMS even in these conditions. Additionally, samples were acquired and analysed with Chemstation software by a human analyst for comparison with the EasyLCMS results.

#### Comparison of standards using agilent Chemstation and EasyLCMS

**Figure 5:**
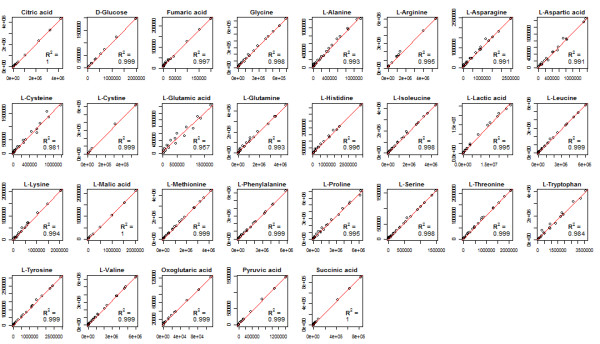
**EasyLCMS performance using calibration standards.** Calibration standard areas obtained using Chemstation software (x-axis) compared with areas obtained using EasyLCMS (y-axis) in all the experiments performed to validate EasyLCMS (see Supplemental Material). Linear regressions (red lines) and regression coefficients (R^2^) are represented for the 29 metabolites analysed.

The metabolite standard areas used for calibration in both platforms were compared for all the analyses performed and detailed in the Supplemental Material (three for amino acids and two for organic acids). The results presented in Figure 5 show a high correlation, R^2^ > 0.99, for almost all of the 29 metabolites analysed. These results demonstrate the strong correlation between both platforms, which was maintained even in experiments separated by lengthy periods.

#### Comparison of samples using agilent Chemstation and EasyLCMS with an internal standard

As described in the Materials and Methods section of the Supplemental Material, six different cultivations (numbers 1 to 6) were performed and three different extraction protocols (ACN, MeOH and Chloro) were followed. From these samples, 21 internal amino acids were analysed.

Globally, 378 concentrations were determined and the results are summarised in Table 1. As can be seen, 93.9% are below 10% relative error, and only 1.6% exceeds 15%, demonstrating the strong reliability of EasyLCMS since a human analyst using Chemstation Software obtained very similar results.

**Table 1:** Distribution of relative error values comparing EasyLCMS and the Chemstation software with an internal standard

**Relative error range**	**Percentage of comparisons**
**0%–5%**	68.8%
**5%–10%**	25.1%
**10%–15%**	4.5%
**15%–23%**	1.6%
**TOTAL**	100%

**Figure 6:**
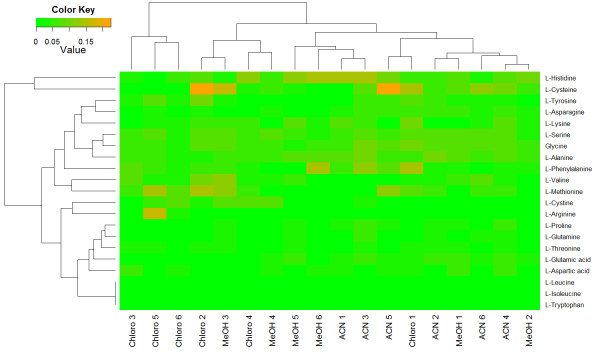
**Two-dimensional clustering representation of relative errors comparing EasyLCMS and Chemstation software with an internal standard.** Twenty-one intracellular amino acids were quantified in samples from six different batch reactors of the human CCL-159 cell line (numbers 1–6), which were obtained with three different extraction procedures (ACN, MeOH and Chloro). See Supplemental Material for details.

Since different extraction methods could have different recovery yields, samples are expected to be clustered by the extraction method. The cluster analysis represented in Figure 6 reveals that of the three extraction procedures, ACN and Chloro presented a different distribution of relative errors, since they were clustered separately with only one exception (Chloro 1), while MeOH displayed an intermediate behaviour, as expected. As regards the metabolites, the highest relative error distributions were shown by L-histidine and L-cysteine. These metabolites deserved a thorough analysis, since they seemed to present recurrent mistakes.

#### Comparison of samples using agilent ChemStation and EasyLCMS without internal standard

For this comparison, two different human cell lines (R, S) were cultured and samples were harvested at six different times (0, 23, 46, 71, 95 and 116 h). From these experiments, 29 internal metabolites were analysed, including amino acids and organic acids. Additionally, the extracellular content of these metabolites was also quantified, obviously without any extraction procedure. For further details, see the Materials and Methods section in the Supplemental Material.

In total, 696 concentrations were determined; the results are summarised in Table 2. As can be seen, 93.2% of the comparisons showed a relative error below 10%, and only 0.6% was over 15% relative error. This confirms the reliability that EasyLCMS showed previously using a different approach and without an internal standard.

**Table 2:** Distribution of relative error values comparing EasyLCMS and Chemstation software without internal standard

**Relative error range**	**Percentage of comparisons**
**0%–5%**	75.7%
**5%–10%**	17.5%
**10%–15%**	6.2%
**15%–22%**	0.6%
**TOTAL**	100%

**Figure 7:**
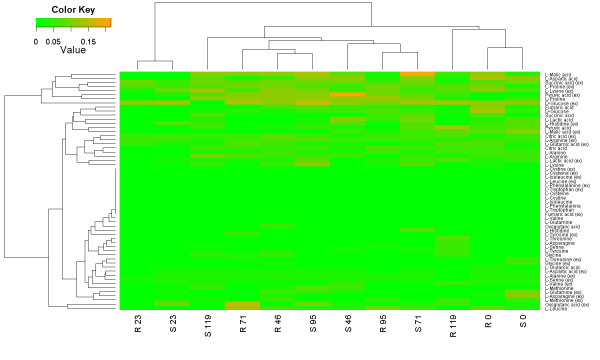
**Two-dimensional clustering representation of relative errors comparing EasyLCMS and Chemstation software without an internal standard.** Twenty-nine metabolites were quantified in samples from two different human cell lines, CCL-159 (S) and CCL-159R (R), which were cultured in batch reactors. Samples were harvested at six different times (0, 23, 46, 71, 95 and 116 h) for extracellular (metabolite names include ‘ex’) as well as intracellular content. See Supplemental Material for details

The cluster analysis is shown in Figure 7. Taking into account sample conditions, initial times (0 and 23 h) are clustered together, probably because the metabolic distributions were similar in the initial stages since growth was initiated with the same medium. The most interesting metabolites were intracellular L-proline, malate and L-aspartate, as well as extracellular D-glucose, L-proline, L-lysine, succinate and pyruvate, since they presented the highest relative error distributions.

## Discussion

### Web platforms vs. software tools in metabolomics data pre-processing

Pre-processing of metabolomics data is carried out in two types of platforms: stand-alone and web applications. Stand-alone applications are software tools which must be installed in a computer and use its resources (memory, processor, etc.) to perform the calculations needed in the data pre-processing; in contrast, web applications are run in generic web browsers with minimal software additions. The use of web applications in data handling could provide the following advantages over stand-alone applications [11]: Firstly, their general use, since they are platform independent and therefore can be applied not only in numerous operating systems, but also in several devices, with only the need for a compatible web browser. In contrast, software tools require a version for every operating system with the exception of Java-based systems. Secondly, minimal installation is needed, as previously described. Thirdly, no installation is needed for fixes or updates in every computer, since web applications are automatically actualised. Fourthly, web applications allow experimental results to be easily available on the web for the scientific community. Fifthly, huge memory and strong processors are required for metabolomics data pre-processing, and consequently, several platforms have been developed for high performance servers or computer clusters [4,26]. Web applications could avoid this investment by centralising computation requirements. In this way, even simpler devices—such as tablets or smartphones, which nowadays are replacing traditional desktop computers— could be used to access the platform. However, desktop applications with massive processing requirements may not be suitable for these devices.

Web applications also have the following drawbacks compared with stand-alone ones: Firstly, desktop applications respond almost instantaneously. However, classical web applications (synchronous communication) require data transfer between the client and the server. Secondly, web applications have a poorer interface than stand-alone ones, since the web page must be completely updated after any modification, which requires massive data transfer. Moreover, real-time interaction is usually avoided, since this requires even higher data transfer. Thirdly, uploading data could compromise the privacy of the experimental results. Although this could be important, open access to raw data files is also appreciated by the science community. In any case, EasyLCMS maintains privacy through user accounts with passwords and anonymous accounts which are completely deleted after 12 hours (including raw data files).

Only a few web applications are available for metabolomics data handling; this is probably due to these drawbacks. For target metabolomics, chromatogram visualisation is highly recommended, and analysts should be able to interact with pre-processing results to avoid mistakes in the automated data handling process. However, even fewer web applications allow chromatogram visualisation; the applications that do allow this include the MetabolomeExpress Project [2] and MeltDB [23] for GC-MS data, as well as the on-line version of XCMS [25] for raw LC-MS data. To our knowledge, EasyLCMS is the only web application able to allow not only chromatogram visualisation, but also interaction using asynchronous web technology. This techno-logy avoids data transfer by reflecting changes instead of reloading the web page completely [12].

### Comparison of application results with manual operation

GC-MS usually requires derivatisation of metabolites, and therefore the use of internal standards is mandatory [28]. In this case, the best option is usually stable isotopes of the analytes to be measured, although these are extremely expensive or not available at all. However, LC-MS does not require derivatisation in most cases, and in consequence, isotopes can be replaced with generic internal standards. In practice, analytical methods developed for LC-MS include a few or only one internal standard for all the assayed metabolites [29,30]. Moreover, metabolite quantification using HPLC-MS has been validated without internal standards [31], although fully labelled cell extracts which incorporate the isotopic internal standard have been highly recommended due to the increment in the precision of the measurements [32]. Therefore, EasyLCMS has been tested to operate correctly using a ‘generic’ internal standard, and even in the extreme situation of using no internal standard.

To validate the quantification procedure of EasyLCMS, raw data from different analytical methods employing HPLC-MS have been processed using Chemstation software and EasyLCMS. When standard mixtures were analysed, manual and EasyLCMS results matched perfectly, even quantifying the same metabolites in different experiments. However, results from more than 1000 concentration comparisons in real samples showed that 94% were below a 10% relative error and 1% was higher than 15% relative error. These results are very satisfactory and in agreement with a previous work, which highlighted the need for better algorithms for alignment, peak detection and deconvolution [20].

The metabolites with the highest relative error distribution were found using clustering analysis and they have been studied to highlight recurrent mistakes. In almost all of them, the metabolites presented small concentrations and the signal was very low, even proximal to the baseline. In this case, two mistakes appeared recurrently: Firstly, alignment algorithms could mismatch the correct peak when chromatograms were very crowded [4]. The tendency was to match small peaks from the baseline whose retention time was closer to the correct position. To avoid this issue, EasyLCMS highlights samples in red if the aligned peak is not the highest in the specified range for the alignment process. This does not mean that the highest peak is always correct, but visual confirmation is needed to check the automatic alignment selection. Secondly, some of the metabolites presented an incorrect separation of the peaks on the deconvolution step or were not even detected. EasyLCMS is able to perform manual integration in order to correct this issue. Obviously, selection of different processing parameters could fix these problems, for example, filtering small peaks by setting a required minimum area. In consequence, the number of peaks would be reduced, and therefore the alignment algorithm could easily retrieve the correct peak, since very small peaks—which probably originate through instrument noise—will not be included in the alignment process. However, additional issues could emerge, for example a lack of detection of interesting peaks.

Since real samples are enormously heterogeneous related to the number of metabolites and their concentrations are in different orders of magnitude, finding parameters with an optimal functioning for every single metabolite could be a hard task, or even one with no solution. Therefore, EasyLCMS allows interaction to correct singular mistakes of the automatic process. To our knowledge, EasyLCMS is a unique web application which is able to integrate (i) automated quantification, (ii) the highlighting of potential alignment mistakes and (iii) analyst interaction. Additionally, the time required differs enormously: With manual integration and using Excel to perform the calibration, several hours are necessary, in contrast to the few minutes needed using EasyLCMS.

### Limitations

The principal limitation is that multiple spectra data (MS/MS or MS^n^) cannot be used at this point, although efforts are being carried out in this direction. Additionally, uploading large files—which usually are produced in LC-MS experiments—could be an issue, and this is even more salient using vendor proprietary data or mzML formats. However, NetCDF and mzXML formats use data compression to reduce the file size [20]. Moreover, zip compression could reduce file size by almost 10 times, even using the NetCDF format.

## Conclusions

EasyLCMS is a web application designed to simultaneously quantify numerous metabolites, taking into account non-lineal retention time drift in LC-MS. Additionally, a rich interface based on asynchronous web technology has been developed, allowing the easy and fast interaction of the analyst with processed raw LC-MS data to check and correct potential mismatching or incorrect peak detection of the automatic algorithms. Several metabolomics platforms require quantified data and metabolite IDs as input, and EasyLCMS can be used as a bridge between raw data and these platforms.

## Availability and requirements

**Project name:** EasyLCMS

**Project home page:**http://www.easylcms.es

**Operating system(s):** Host server equipped with two Intel Quad Core 2 processors (2 GHz each) and 8 GB of physical memory running the Windows Server 2008 R2 (64-bit) operating system.

**Programming language:** Microsoft .NET and Microsoft Silverlight 4.0

**Other requirements:** For Windows and Mac operative systems, Silverlight, a free Microsoft plug-in (http://www.silverlight.net) is required. For Linux, a compatible interface has been developed and Silverlight is not needed. EasyLCMS has been tested in the principal navigators (Internet Explorer 9.0, Firefox 4.0 and Google Chrome).

**License:** Free to use for academic users.

**Any restrictions to use by non-academics:** Licence needed.

## Availability of supporting data

The dataset supporting the results of this article is included within the article (and its additional file).

## Abbreviations

LC: Liquid chromatography; NMR: Nuclear magnetic resonance; GC-MS: Gas chromatography–mass spectrometry; CE-MS: Capillary electrophoresis–mass spectrometry; LC-MS: Liquid chromatography–mass spectrometry; LC-UV: Liquid chromatography–ultraviolet; MS: Mass spectrometry.

## Competing Interests

The authors declare that they have no competing interests.

## Authors' contributions

SF developed the web application. AS designed the web application and wrote the manuscript. CB and ABL performed the experimental work and tested the web application. JLI and MC participated in the design and coordination of the study and revised the manuscript critically. All authors read and approved the final manuscript.

## Funding

This work was supported by BIO2008-045-C02-01 and BIO2011-29233-C02-01, Séneca project 08660/PI/08, NEOTEC (CDTI, Spain), CARM (Murcia, Spain) and INFO (Murcia, Spain); MCINN and FSE (PTQ and PTA Subprograms).

## Acknowledgements

We thank Inbionova Biotech S.L. for hosting EasyLCMS.
